# Progesterone Receptor (PGR) gene polymorphism is associated with susceptibility to preterm birth

**DOI:** 10.1186/s12881-015-0202-1

**Published:** 2015-08-19

**Authors:** Immaculate Mbongo Langmia, Yamunah Devi Apalasamy, Siti Zawaih Omar, Zahurin Mohamed

**Affiliations:** Department of Pharmacology, Pharmacogenomics Laboratory, Faculty of Medicine, University of Malaya, 50603 Kuala Lumpur, Malaysia; Department of Obstetrics and Gynecology, University of Malaya, 50603 Kuala Lumpur, Malaysia

**Keywords:** Genetic polymorphisms, Preterm birth, Progesterone receptor, Susceptibility, Ethnicity

## Abstract

**Background:**

Preterm birth (PTB) is the major cause of death in newborn and the second major cause of death in children less than 5 years old worldwide. Genetic polymorphism has been implicated as a factor for the occurrence of preterm birth. The aim of this study is to evaluate whether polymorphism in the progesterone receptor (PGR) is associated with susceptibility to preterm birth.

**Methods:**

A total of 135 women with preterm and 532 women with term deliveries were genotyped for PGR gene polymorphisms (rs660149, rs471767, rs10895068) using Sequenom MassARRAY platform.

**Results:**

The G allele of PGR rs660149 polymorphism was significantly associated with susceptibility to PTB in the Malay women. The odds of G allele occurring among Malay women with preterm delivery was twice that of Malay women with term delivery (OR 2.3, 95 % CI (1.2–4.5, P = 0.011). Alternatively, no significant association was observed between PGR rs660149 polymorphisms and susceptibility to PTB in Chinese and Indian women.

**Conclusions:**

This study shows that variability in the occurrence of PTB across ethnicities in Malaysia is partly due to differences in genetic background. We therefore suggest that in addition to life style and environmental factors, genetic factor should be greatly considered in this population. Prior information on the genetic composition of women may help in the identification and management of women at risk of preterm birth complication.

## Background

Preterm birth is the major cause of death in the newborn and the second major cause of death in children less than 5 years old worldwide [1]. Approximately 1 in 10 babies are born preterm [1]. There are two main types of preterm birth, medically indicated preterm birth which occurs due to induction of labor as an intervention to save the mother or child especially during pregnancy complications such as preeclampsia, gestational diabetes, fetal distress, renal disease, unexplained vagina bleeding, Rh sensitization, congenital malformation, placenta previa and abruption of placenta [2–5]. Otherwise spontaneous preterm birth (sPTB) which is the most predominant type of PTB occurs due to a sudden onset of labor [1, 6]. The aetiology of spontaneous preterm birth is multifactorial and still remains largely unknown [7]. Preterm infants have high risk of life-threatening health problems such as bronchopulmonary dysplasia, hypotension, intracerebral haemorrhage, anemia, jaundice, hearing loss, and retinopathy of prematurity [8]. According to the World Health Organization (WHO), the global health progress in child survival can only be achieved if preterm birth is properly addressed [1]. The prevalence of preterm birth vary among ethnic groups and this variability has been attributed to genetic and environmental factors with maternal genetic factors contributing up to 20 % risk for developing PTB [9, 10]. One study carried out in the American population has revealed that genetic disparity between the Caucasians and African Americans could explain the differences in the incidence of preterm birth between the two ethnic groups [11, 12]. Malaysia is a multi-ethnic country consisting of three major ethnic groups, namely the Malays, Chinese and Indians, each of which are of different genetic background, thus presenting a good opportunity to study the genetic disparity of preterm birth with regards to PGR gene polymorphism. In this study we aim to explore the genetic influence of PGR gene polymorphisms on the risk of PTB among Malaysian women.

Progesterone (PG) is a major female ovarian hormone needed for successful pregnancies in women [13–15]. During pregnancy, the uterus is maintained in a quiescent state by balanced proportion of various compounds including progesterone, prostacyclin, nitric oxide, relaxin and parathyroid hormone related peptide [16]. The binding of progesterone to the progesterone receptor (PGR) is required for PG function and a successful establishment and maintenance of pregnancy [17]. In animal studies, removal of progesterone results in inability to establish pregnancy [18]. Yellon et al. 2013 showed that the termination of progesterone receptor-mediated actions led to the structural remodeling of the cervix, increased macrophage production, and premature birth in preterm cervix compared to term cervix. Thus mothers with disrupted progesterone receptor-mediated function are likely to deliver their babies much sooner than expected. Pharmacogenetic variation in the progesterone receptor gene alters progesterone function resulting in initiation of preterm labor/birth [19]. We aim to investigate the impact of PGR (rs660149, rs10895068 and rs471767) gene polymorphisms on the risk of PTB in Malaysia.

## Methods

### Study subjects

The study involved a total of 667 women (135 preterm and 532 term) with spontaneous singleton delivery admitted at the University of Malaya Medical Centre (UMMC) from 2011 to 2013 respectively. Subjects were classified into two groups; the preterm group consisted of mothers who delivered their baby between 24 and 36 weeks while the control group consisted of mothers with uncomplicated pregnancy who delivered their babies between 38 and 41 weeks. Prior to sample collection, each participant was allocated a written informed consent form, patient information sheet and a data collection sheet. The data collection sheet consisted of all information about the patient’s demographic, obstetric history, life style, and family history. Ethnicity of the participants was obtained from the data collection sheet in which participants self reported their ethnicities as Malays, Chinese or Indians and declared that there has been no mixed marriage for at least three generations. The study was approved by the University of Malaya Medical Centre (UMMC) Ethics Committee and written informed consent was obtained from all participants.

Trained gynecologists assessed the physical well-being of the participants and pregnancy age was calculated from the first day of the last menstrual cycle or obtained from ultrasound results of the patients. The following inclusion criteria was used to select the participants; healthy mothers between the ages of 18–35 years old, mothers with normal competent cervix and uterus, mothers without metabolic or autoimmune disease and mothers who conceived preterm but with normal and healthy fetus. Exclusion criteria included abnormal fetus and still birth, mothers pregnant with twins, birth delivery by induction due to fetal distress or placenta abruption or preeclampsia or hypertension or any known medical problem were excluded, maternal age of less than 18 years or more than 35 years, mothers with history of drug abuse, and mothers who were not able to sign informed consents were also excluded from the studies. Medical records of the participants were reviewed to obtain more patient details.

### SNP genotyping

A volume of 3mls of maternal venous blood was collected in to EDTA tubes and centrifuged at 2000 × g for 10 minutes. The buffy coat and plasma were kept in separate 1.5 ml tubes and stored in the freezer until utilized. Maternal genomic DNA was extracted from buffy coat using the GeneAll^@^ Exgene^TM^ DNA purification kit. Three SNPs in the PGR gene (rs660149C/G, rs10895068A/G and rs471767A/G) were genotyped using Sequenom MassARRAY platform. SNPs with call rate of greater than 90 % were considered for statistical analysis. For the three PGR SNPs that were genotyped, two (PGR rs660149 and rs471767) with call rates of 99 % were considered for statistical analysis while the PGR rs10895068 SNP with a call rate of 87 % was excluded from statistical analysis.

### Statistical analysis

Statistical analysis of subjects was performed using SPSS version 18.0 (IBM Corp., Chicago, IL, USA). Prior to genetic analysis, Hardy-Weinberg equilibrium (HWE) calculator was used to check for deviation from HWE using a goodness of fit χ^2^ test. The association and allele-based test for PGR rs660149 and rs471767 with preterm birth was performed using Fisher’s exact test with 2 and 1 degrees of freedom respectively. Pearson’s χ^2^ test or independent t-test was used to compare categorical and continuous variables between groups and data were presented as percentage or mean ± standard deviation. Multivariate analysis was performed with adjustment for a past history of PTB, gestational diabetes and miscarriage respectively. It should be noted that confounding factors such as smoking, substance abuse, mothers who were themselves born preterm and alcohol consumption were not included for statistical analysis because all subjects were tested and found to be free from these factors. The Quanto version 1.2.4 soft ware was used to calculate power of the study.

## Results

The demographic and clinical parameters of subjects are presented in Tables 1 and 2. Significant differences were observed in past history of preterm birth, miscarriage and gestational diabetes in overall subjects. Following stratification by ethnicity, significant findings were observed for past history of preterm, GDM and miscarriage in Malay ethnic subgroup (Table 2). Significant differences were also seen for past history of PTB and GDM in Indians and Chinese ethnic subgroups respectively (Table 2). Alternatively, there were no significant differences in maternal age, BMI, income and marital status between preterm and term groups in overall subjects as well as in the Malays, Indians and Chinese ethnic groups (Table 2).

**Table 1:** Demographic features of 532 term and 135 preterm subjects

Characteristics	Term	Preterm	*p* value
Maternal age	29.3 ± 3.7	29.8 ± 3.0	0.23
Maternal BMI (kg/m2)	24.5 ± 5.4	24.3 ± 5.1	0.69
Married subjects (%)	98	98	0.72
Gestational diabetes (%)	0	11	**0.000**
History of PTB (%)	0	14	**0.000**
Previous miscarriage (%)	15	25	**0.011**
Low income (%)	2	3	
Average income (%)	33	35	0.561
High income (%)	65	62	

Data are express as mean ± SD for continuous data and as percentage for categorical data

p values were obtained using Mann–Whitney U test or independent t test

*P*-values <0.05 were considered significant

**Table 2:** Demographic characteristics of subjects by ethnicity

Characteristics	Malays (*N* = 480)			Indians (*N* = 94)			Chinese (*N* =93)		
	Term (*N* = 388)	Preterm (*N* = 92)	*P* value	Term (*N* = 74)	Preterm (*N* = 20)	*P* value	Term (*N* = 70)	Preterm (*N* = 23)	*P* value
Maternal age	29.2 ± 5.6	29.8 ± 4.1	0.249	30.0 ± 3.7	30.3 ± 3.7	0.938	25.9 ± 3.6	29.4 ± 3.5	0.946
Maternal BMI (kg/m2)	24.3 ± 5.6	24.6 ± 5.4	0.267	25.6 ± 5.7	24.2 ± 5.5	0.550	23 ± 5.5	24.8 ± 4.5	0.329
Married subjects (%)	100	99	0.974	98	99	0.961	100	99	0.920
Gestational diabetes (%)	0	8.0	0.000	0	10	0.004	0	20	0.000
History of PTB (%)	0	14	0.000	0	15	0.000	0	12	0.005
Previous miscarriage (%)	14	24	0.017	19	26	0.511	17	28	0.288
Low income (%)	2.8	3.0		3	5		1	0	
Average income (%)	29	35	0.212	55	31	0.161	27	36	0.593
High income (%)	68	61		41	63		72	64	

Data are express as mean ± SD for continuous data and as percentage for categorical data

P values were obtained using Mann–Whitney U test or independent t test

*P*-values <0.05 were considered significant

The allelic association test between PGR rs660149 SNP and preterm birth in the overall subjects and in various ethnic subgroups are shown in Table 3. There was no deviation from Hardy Weinberg equilibrium for PGR rs660149, rs471767 in preterm and term groups both in overall subjects and after stratification by ethnicity. The G allele of PGR rs660149 was associated with susceptibility to preterm birth in overall subjects (OR, 1.8, 95 % CI, 1.0–3.1) *p* = 0.042). Following stratification by ethnicity, the G allele of PGR rs660149 was significantly associated with susceptibility to preterm birth in the Malay ethnic subgroup. The odds of the G allele occurring among women with preterm birth was twice that of women with term birth (Odd ratio 2.3, 95 % CI 1.2–4.5, *P* = 0.011). We observed no significant association between the G allele of PGR rs660149 and susceptibility to preterm birth in Chinese and Indian ethnic groups (Table 3). The allelic frequency of the PGR rs471767 polymorphism was not significantly associated with susceptibility to PTB in overall subjects and as well as after stratification by ethnicity (Table 3). There was a significant difference in the rs660149 genotype distribution between preterm and term groups in the Malays (*P* = 0.014). The heterozygous CG and homozygous GG genotypes of rs660149 were high among Malay women with preterm delivery compared to term (Table 4). The genotype distribution of the rs660149 polymorphism was not different between women with preterm and term delivery in the Indians and Chinese ethnic groups. Likewise there was no significant association seen in the genotype distribution of rs471767 polymorphism in all three ethnic groups (Table 4). Results from quanto soft ware analysis yielded a power of 86 %, based on the following criteria; sample size of 135 patients, for allele frequency ranging from 0.05 to 0.12, a population risk of 0.17, with the minimum detectable odds ratio of 1.8.

**Table 3:** Association test of PGR rs660149 and rs471767 in pooled subjects, Malays, Indians and Chinese ethnic groups

Ethnicity/SNPs	Alleles frequencies		Adj-OR (CI)	Adj-P
	Preterm versus term			
PGR, rs660149C/G	C	G		
Overall	0.93 vs 0.96	0.07 vs 0.04	1.8 (1.00 - 3.1)	0.041
Malays	0.91 vs 0.96	0.09 vs 0.04	2.3 (1.20 - 4.5)	0.011
Indians	0.95 vs 0.93	0.05 vs 0.07	1.3 (0.26 - 6.4)	0.758
Chinese	0.93 vs 0.09	0.07 vs 0.91	1.1 (0.20 - 6.2)	0.891
	Preterm versus term			
PGR, rs471767A/G	A	G		
Overall	0.89 vs 0.88	0.12 vs 0.12	1.0 (0.68 – 1.6)	0.884
Malays	0.89 vs 0.96	0.11 vs 0.12	1.0 (0.53 - 1.5)	0.703
Indians	0.82 vs 0.86	0.18 vs 0.14	1.4 (0.56 – 3.7)	0.447
Chinese	0.89 vs 0.92	0.11 vs 0.08	1.1 (0.20 - 6.2)	0.891

Significant p-value, P computed using multiple regression analysis

P values were adjusted for previous PTB, previous miscarriage, infection and gestational diabetes

*Adj-P* adjusted P value

**Table 4:** Genotype association test of PGR rs660149 and rs471767 SNPs

SNP	Genotype frequency			P
Preterm vs Term				
rs660149C/G	CC	CG	GG	
Overall	0.85 vs 0.92	0.15 vs 0.08	0.00 vs 0.00	0.074
Malays	0.73 vs 0.92	0.17 vs 0.07	0.10 vs 0.05	**0.014**
Indians	0.89 vs 0.87	0.11 vs 0.13	0.00 vs 0.00	0.757
Chinese	0.08 vs 0.07	0.92 vs 0.93	0.00 vs 0.00	0.891
rs471767A/G	AA	AG	GG	
Overall	0.78 vs 0.77	0.21 vs 0.21	0.02 vs 0.01	0.546
Malays	0.79 vs 0.76	0.20 vs 0.23	0.01 vs 0.00	0.812
Indians	0.68 vs 0.74	0.26 vs 0.24	0.05 vs 0.01	0.557
Chinese	0.83 vs 0.87	0.13 vs 0.10	0.04 vs 0.03	0.891

P: Chi square *P* value, *P* < 0.05 was considered significant

## Discussion

Progesterone plays an important role in the maintenance of pregnancy up to when the baby is fully mature and ready for parturition [17]. The role of progesterone in pregnancy is inevitable as research have shown that ablation of progesterone results in a sudden uterine contraction and preterm birth [17–18, 20–21]. Preterm birth involves a change of the state of the uterus from a quiescent to a contractile state; this change is prevented by progesterone which exerts its function by binding to its receptor. Progesterone treatment has been useful in preventing preterm birth [17, 20, 22–24]. It has been suggested that genetic variation that might result in alterations of the biologic functions of progesterone receptor can possibly contribute to susceptibility to preterm birth [19, 25].

In this study we showed a significant association of PGR rs660149 G variant with susceptibility to preterm birth in the Malay ethnic group. Subjects with the G allele were two times more likely to experience preterm birth than control subjects. It should be noted that the Malays form the largest community in Malaysia and represent more than half of the population. This result is similar to the result of Ehn et al. in which they found associations between PGR SNPs and susceptibility to preterm birth [19].

The significant results obtained after adjustment for confounding factors is an indication that genetic polymorphism is a major risk factor of preterm birth complication in this population. Prior information on the genetic composition of women with spontaneous preterm birth may help in the identification and management of high risk women. Malaysia has a multiethnic population which is composed of three different ethnic subgroups namely Malay, Chinese and Indians with varying genetic background. The present study shows that variability in the occurrence of preterm birth among women in Malaysia is due to differences in their genetic components. In addition to the life style and environment factors in this population, genetic factors play a major role in the disparity of preterm birth amongst ethnic groups. Infants born preterm suffer from major health problems therefore there is a high demand for ethnicity specific genetic and environmental strategies to be put in place to prevent or better manage high risk women in every geographical area. This will enhance survival of neonates who might suffer or die due to preterm birth associated complications.

## Conclusions

In summary, we showed a significant association between PGR rs660149 polymorphism and susceptibility to PTB in the Malay ethnic group. This finding may have implication for screening and treatment of women at risk of preterm birth complication.
